# 非小细胞肺癌组织起源分子病理分类及其临床意义

**DOI:** 10.3779/j.issn.1009-3419.2018.07.05

**Published:** 2018-07-20

**Authors:** 娜娜 王, 楠 江, 晓庆 杨, 磊 房, 青 孙

**Affiliations:** 1 250000 济南，山东大学附属千佛山医院病理科 Department of Pathology, Qianfoshan Hospit al Affiliated to Shandong University, Jinan 250000, China; 2 250022 济南，山东省立医院西院病理科 Department of Pathology, Shandong Provincial Hospital West Hospital, Jinan 250022, China

**Keywords:** 肺肿瘤, 组织起源, 病理分类, EGFR, Lung neoplasms, Histogenesis, Pathological classification, EGFR

## Abstract

**背景与目的:**

近年来对肺癌研究的巨大发展，肺癌的病理学分类不断推陈出新。本研究旨在研究和验证非小细胞肺癌（non-small cell lung cancer, NSCLC）组织起源分子病理分类的临床价值和意义。

**方法:**

通过免疫组织化学双染法对105例肺癌标本及正常肺组织进行P63/NapsinA、TTF-1/CK7标记，结合肿瘤免疫特征、组织学特点及正常肺组织的免疫特征，提出了NSCLC组织起源分子病理分类体系，将NSCLC分为支气管上皮癌、细支气管肺泡癌、肺泡细胞癌及分泌腺癌，并分析本分类与表皮生长因子受体（epidermal growth factor receptor, *EGFR*）突变及临床预后的关系。

**结果:**

本分类体系下不同亚型NSCLC中*EGFR*突变及其类型具有相对特异性；生存分析表明该分类有助于NSCLC患者预后情况的判断。

**结论:**

本研究提出的基于组织起源的NSCLC分类体系具有较好的临床实用价值。

## Abstract (en)

**Background and objective:**

Due in part to remarkable advances over the past decade in our understanding of lung cancer, there is a pressing need for a revised classification. The aim of this study was to explore and verify the clinical significance of a new molecular classification of non-small cell lung cancer (NSCLC) on the basis of anatomic site of bronchial tree.

**Methods:**

Double-immunohistochemistry were performed in 105 tumor samples paring with normal lung tissue from non-small cell lung cancer patients by using monoclonal antibody of P63/NapsinA and TTF-1/CK7. By comprehensively analyzing the express profiles of tumors and normal lung tissues, histological characteristics we proposed a brand new pathological classification based on histogenesis for NSCLC and divided them as: bronchiole epithelial cell carcinoma, bronchiole-alveolar cell carcinoma, alveolar cell carcinoma and secretory adenocarcinoma. And to analyze the relationship between this classification and epidermal growth factor receptor (*EGFR*) mutation and ognosis.

**Results:**

Further investigation revealed that our new classification showed strong relevancy with *EGFR* mutations and effective indicators for prognosis.

**Conclusion:**

The classification system for NSCLC proposed by our research group is convenient for pathological diagnosis and valuable in clinical application.

肺癌是目前世界范围内死亡率最高的恶性肿瘤^[1]^，其中非小细胞肺癌（non-small cell lung cancer, NSCLC）约占其总数的85%^[2-4]^，成为严重威胁人们生命健康的社会问题。现有的NSCLC病理分类依据是建立在形态和结构基础上的2015版世界卫生组织（World Health Organization, WHO）肺部肿瘤的组织病理学分类，由于肺组织本身结构复杂，癌组织又存在不同程度的异质性，在临床病理诊断中往往会遇到部分含混不清且难以分辨者，直接影响治疗方案的正确选择。本研究从组织起源的角度对NSCLC进行表型分化分类，结合其表皮生长因子受体（epidermal growth factor receptor, *EGFR*）突变检测和预后分析，力求使目前的NSCLC组织病理学分类更加清晰明了，更贴近指导临床应用。

## 1 材料与方法

### 1.1 临床资料

收集山东大学附属千佛山医院病理科自2010年1月-2013年12月NSCLC患者石蜡包埋组织标本共105例。依据2015年WHO肺癌诊断标准，经两名经验丰富的病理诊断医师对全部切片进行病理分类，挑选同时具有正常肺组织和癌组织的切片及对应蜡块，为本研究所用；所有患者术前均未接受放疗及化疗。其中男性76例，女性29例。 < 61岁者52例，≥61岁者53例。肿瘤直径≤3 cm者38例， > 3 cm者67例。有淋巴结转移者71例、无淋巴结转移者34例。本研究已通过山东大学附属千佛山医院伦理委员会审查并批准。

### 1.2 免疫组织化学双染色

#### 1.2.1 仪器及试剂

Bench Mark全自动免疫组化染色机（美国Roche公司）；TTF-1鼠抗人抗体（Mab-0599，迈新公司），P63鼠抗人抗体（Mab-0365，迈新公司），Napsin A兔抗人抗体（LBP-m198，安必平公司），CK7鼠抗人抗体（LBP-mo61，安必平公司）；ultraView Red二抗试剂盒（美国Roche公司）。

#### 1.2.2 染色步骤

预先对免疫组化仪进行编程，使用P63/NapsinA组合，TTF-1/CK7组合。组合抗体中前者使用DAB显色，染色呈棕色；后者使用碱性品红染液显色，染色呈红色。

#### 1.2.3 结果判定

NapsinA、CK7阳性表达位于细胞质，染色呈红色；P63、TTF-1阳性表达位于细胞核，染色呈棕褐色。着色即判为阳性，散在点灶状阳性、弥漫阳性、弥漫强阳性均视为阳性表达，不着色则判为阴性。

### 1.3 SANGER测序法检测*EGFR*基因突变

常规提取石蜡组织内DNA，以设计引物进行PCR扩增。使用下述各突变引物序列进行扩增：EGFR第18号外显子正向引物：5'-CAAATGAGCTGGCAAGTGCCGTGTC-3'，反向引物：5'-GAGTTTCCCAAACACTCAGTGAAAC-3'；EGFR第19号外显子正向引物：5'-GCAATATCAGCCTTAGGTGCGGCTC-3'，反向引物：5'-CATAGAAAGTGAACATTTAGGATGTG-3'；EGFR第20号外显子正向引物：5'-CCATGAGTACGTATTTTGAAACTC-3’，反向引物：5'-CATATCCCCATGGCAAACTCTTGC-3'；EGFR第21号外显子正向引物：5'-CTAACGTTCGCCAGCCATAAGTCC-3’，反向引物：5'-GCTGCGAGCTCACCCAGAATGTCTGG-3’。PCR反应体系为25 μL：模板DNA 1 μL；正反向引物各0.5 μL；10×PCR Buffer 5 μL；dNTP Mix 2 μL；StarTaq DNA Polymerase 0.25 μL；Distilled water 15.75 μL。PCR反应条件：置PCR扩增仪中95 ℃预变性15 min，94 ℃变性50 s，62 ℃退火1 min，72 ℃延伸1 min，共循环10次，94 ℃变性50 s，57 ℃退火1 min，72 ℃延伸1 min，共循环30次，最后于72 ℃延伸10 min。使用美国Applied Biosystem公司3150型基因序列分析仪进行检测，应用Applied Biosystem公司Data Collection与Sequencing Analysis软件进行数据分析。

### 1.4 统计方法

肿瘤表达指标与临床病理参数的联系采用卡方检验和*Fisher*精确概率法；单因素生存分析采用*Kaplan-Meier*法；所有数据采用SPSS 18.0（SPSS, Chicago, IL, USA）统计软件进行统计学分析，以*P* < 0.05为差异具有统计学意义。

## 2 结果

### 2.1 P63/NapsinA、TTF-1/CK7在正常肺组织的定位及其表达

CK7、NapsinA阳性染色呈红色，位于细胞浆；P63、TTF-1阳性染色呈棕黄色/棕褐色，位于细胞核。P63表达于所有支气管呼吸上皮的基底细胞和分泌腺的肌上皮细胞，而肺泡上皮细胞不表达；CK7表达于所有支气管呼吸上皮细胞（假复层柱状纤毛上皮、单层柱状上皮）、分泌腺上皮细胞及肺泡上皮细胞；TTF-1主要表达于终末小支气管上皮细胞和肺泡上皮细胞。NapsinA仅表达于肺泡上皮细胞。

### 2.2 P63/NapsinA、TTF-1/CK7在NSCLC中的表达

P63阳性表达于鳞癌、贴壁状腺癌、腺泡状腺癌和实体状腺癌（依据2015年WHO肺癌分类）中，阳性表达率分别为100%（41/41）、47.37%（6/13）、53.65%（22/41）、80%（8/10）；CK7阳性表达在贴壁状腺癌、腺泡状腺癌和实体状腺癌中，阳性表达率为100%，鳞癌中未见表达；TTF-1阳性表达于贴壁状腺癌、腺泡状腺癌和实体状腺癌中，阳性表达率分别为84.61%（11/13）、88.89%（36/41）、70.00%（7/10），鳞癌中未见表达；NapsinA阳性表达在贴壁状腺癌、腺泡状腺癌和实体状腺癌中，阳性表达率分别为15.38%（2/13）、78.95%（32/41）和60%（6/10），鳞癌中未见表达。

结合免疫表型特征、组织学形态，我们发现NSCLC与相对应解剖部位的正常组织免疫表型具有一致性，双染免疫组化染色图片见图 1。单纯的鳞状细胞癌P63呈阳性表达，而CK7、TTF-1、NapsinA阴性；单纯腺癌：CK7阳性，TTF-1部分阳性，部分阴性，而P63和NapsinA阴性；支气管发生的鳞腺混合型癌：P63和CK7弥漫阳性，NapsinA阴性，而TTF-1部分病例阳性，部分病例阴性；肺泡细胞癌：CK7、TTF-1和NapsinA阳性，而P63阴性；细支气管肺泡癌：CK7、TTF-1、NapsinA阳性，P63散在阳性，105例NSCLC免疫组化结果见表 1。由此可见支气管、肺泡单位正常组织和对应部位的肺癌具有表型的一致性，细支气管肺泡癌则同时具有细支气管腺癌和肺泡细胞癌的共同特点，呈现一种过渡性表型特征，见表 2。

**1:**
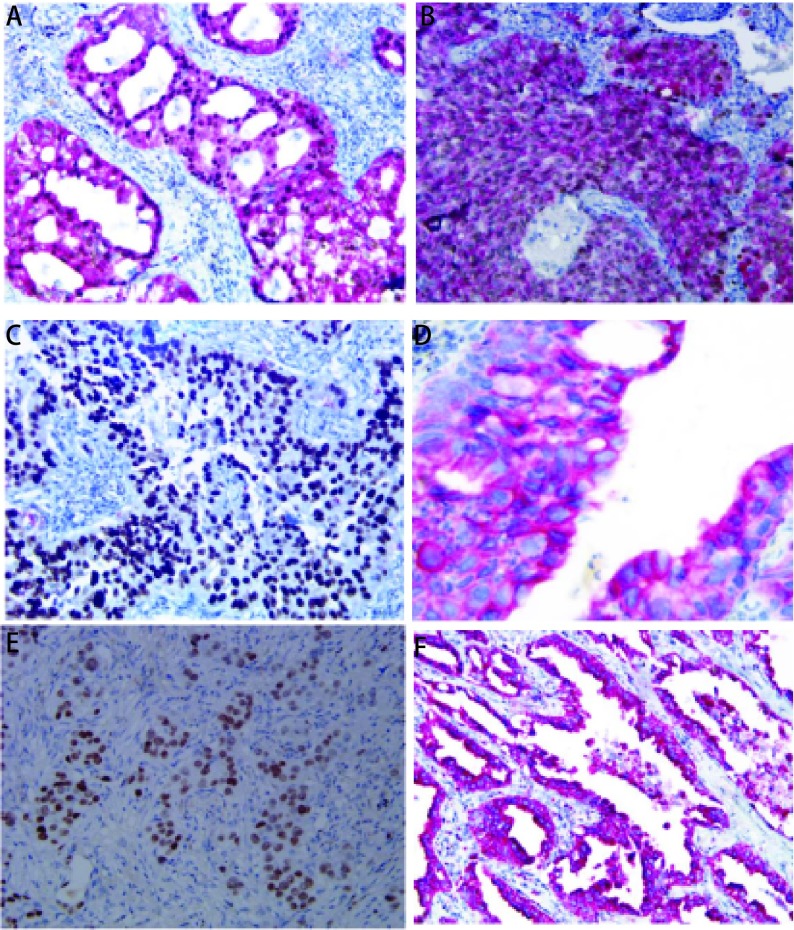
P63、NapsinA、CK7、TTF-1蛋白在NSCLC中的表达。A：CK7 (+)，TTF-1 (+)，(SP, ×200)；B：P63 (+)，NapsinA (+) (SP, ×200)；C：P63 (+)，NapsinA (-) (SP, ×200)；D：CK7 (+)，TTF-1 (-) (SP, ×200)；E：CK7 (-), TTF-1 (+)；F：P63 (-), NapsinA (+) (SP, ×200)。 The expression of P63, NapsinA, CK7 and TTF-1 in NSCLC. A: CK7 (+), TTF-1 (+), (SP, ×200); B: P63 (+), NapsinA (+) (SP, ×200); C: P63 (+), NapsinA (-) (SP, ×200); D: CK7 (+), TTF-1(-) (SP, ×200); E: CK7 (-), TTF-1 (+); F: P63 (-), NapsinA (+) (SP, ×200).

**1:** P63、CK7、TTF-1、NapsinA在肺癌组织中的表达 The expression of p63、CK7、TTF-1、NapsinA in lung cancer tissue

Classification	P63	CK7	TTF-1	NapsinA	*n*
Bronchiole-alveolar cell carcinoma	+	+	+	+	44
Alveolar cell carcinoma	-	+	+	+	11
Adenocarcinoma	-	+	+	+	2
Glandscale cancer	+	+	-	-	37
Squamous carcinoma	+	-	-	-	11

**2:** 正常肺组织及不同组织起源NSCLC免疫表型的关系 The relationship between normal lung tissue and NSCLC immune phenotypes in different tissues

Pathological types	Immunophenotype	P63	CK7	TTF-1	NapsinA
Normal lung tissue	Bronchial respiratory epithelium	+	+	-	-
	Bronchial epithelium	+	+	+	-
	Alveolar epithelial	-	+	+	+
	Secretory gland cell	+	+	-	-
Bronchiole epithelial cell carcinoma	Squamous carcinoma	+	-	-	-
	Adenocarcinoma	-	+	±	-
	Glandscale cancer	+	+	±	-
Bronchiole-alveolar cell carcinoma		+	+	+	+
Alveolar cell carcinoma		-	+	+	+
Secretory adenocarcinoma		±	+	-	-

据此，我们将NSCLC分为以下几种组织表型，即支气管上皮癌（起源于各级支气管，分为单纯鳞癌、单纯腺癌和腺鳞癌）、细支气管肺泡癌（起源于终末细支气管肺泡单位）、肺泡细胞癌（起源于肺泡上皮）、分泌腺癌（起源于支气管粘膜下小唾腺）。本研究中仅有2例小涎腺来源的腺癌，其免疫表型为CK7阳性，P63阳性1例，阴性1例，虽然在免疫表型上与支气管腺鳞癌相同，但由于具有特殊的组织形态，两者鉴别并不困难。

依据依据2015年WHO肺癌诊断标准，将105例NSCLC分为鳞癌、贴壁状腺癌、腺泡状腺癌和实体状腺癌，与105例NSCLC组织表型分类对应见表 3。

**3:** 两种肺癌组织学分类方法之间的对应 The correspondence between two types of histological classification

Classification	Bronchiole-alveolar cell carcinoma	Alveolar cell carcinoma	Adenocarcinoma	Glandscale cancer	Squamous carcinoma	Total
Lepidic	5	7	1			13
Acinar	36	3		2		41
Solid	3	1	1	5		10
Squamous-carcinoma				30	11	41
Total	44	11	2	37	11	105

### 2.3 不同组织起源NSCLC与*EGFR*基因突变的相关性

*EGFR*基因突变在支气管上皮癌、细支气管肺泡癌及肺泡细胞癌中的检出率分别为1.60%（8/50）、52.30%（23/44）、45.50%（5/11），差异有统计学意义（*P* < 0.001）。本研究中不同起源的NSCLC中*EGFR*突变位点呈现不同的特点：支气管上皮癌中有*EGFR*18和21外显子突变；肺泡细胞癌中检测到*EGFR* 20和21外显子突变，未发现18和19外显子的突变；相比之下，细支气管肺泡癌中*EGFR*突变呈现多样性，4种常见突变均被检测到；在支气管上皮癌中仅腺鳞癌表达，差别有统计学意义（*P* < 0.001），见表 4。

**4:** 不同组织起源NSCLC与*EGFR*基因突变相关性 The correlation between NSCLC and *EGFR* gene mutation

Histogenesis for NSCLC		*EGFR* mutation				Mutation type			
		Mutant	Wild type	Mutation rate		18 exon	19 exon	20 exon	21 exon
Bronchiole epithelial cell carcinoma	Squamous carcinoma	0	11	0.00%		0	0	0	0
	Adenocarcinoma	0	2	0.00%		0	0	0	0
	Glandscale cancer	8	29	21.60%		2	0	0	6
Bronchiole-alveolar cell carcinoma		23	21	52.30%		2	8	3	7
Alveolar cell carcinoma		5	6	45.50%		0	0	2	3

### 2.4 不同组织起源NSCLC生存分析

本研究共随访患者105例，失访患者共19例。单因素生存结果分析显示，支气管上皮癌、细支气管肺泡癌和肺泡细胞癌总体预后生存差别无显著意义，当把临床分期作为分层因素时，在Ⅰ期和Ⅱ期肺泡细胞癌预后优于细支气管肺泡癌，而支气管上皮癌预后最差，虽然*P*值均未达到统计学意义上的显著差异（*P*_1_=0.009, *P*_2_=0.075），但其差异已经趋于显著（Ⅲb期患者分组存在较大偏倚性，拟扩大样本量后进一步分析），见表 5、图 2。

**5:** 不同组织起源NSCLC单因素生存分析 Analysis of the existence of NSCLC single factor for different tissue origin

Index	Classification	*n*	Mean survival time (mo)	95%CI	*P*
Histological classification	Bronchiole epithelial cell carcinoma	57	32	27.3-36.5	0.145
	Bronchiole-alveolar cell carcinoma	22	37	32.5-40.1	
	Bronchiole-alveolar cell carcinoma	25	50	47.0-58.6	
Differentiation	Well	35	33	28.0-37.3	0.326
	Moderate	37	29	25.6-32.1	
	Poor	32	26	24.8-30.4	
Tumor size	≤3 cm	38	41	30.6-43.3	0.248
	> 3 cm	68	22	13.4-31.7	
Lymph node metastasis	Yes	72	18	14.2-23.5	0.001
	No	32	35	38.1-47.6	
Clinical stage	Ⅰ	28	52	46.8-55.5	0.050
	Ⅱ	41	35	26.8-40.5	
	Ⅲb	35	19	15.8-25.5	

**2:**
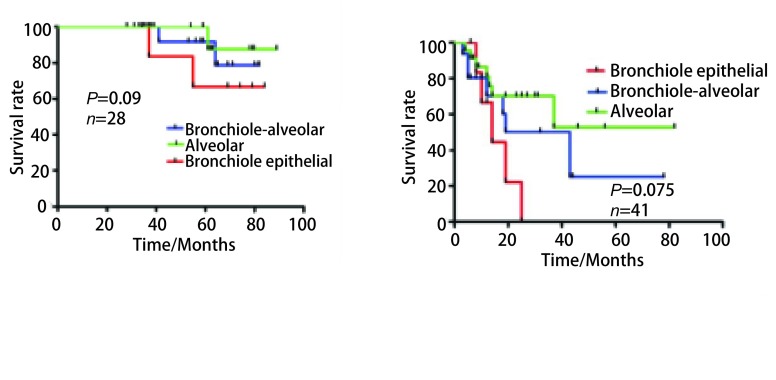
不同临床分期支气管上皮癌、细支气管肺泡癌和肺泡细胞癌生存曲线 The survival curves of bronchial epithelial carcinoma, bronchial alveolar carcinoma and alveolar cell carcinoma

## 3 讨论

以组织学为基础的WHO肺及胸膜肿瘤分类是目前临床广泛采用的肺癌分类体系，尤其是2015年版WHO肺癌分类^[5, 6]^整合了放射、内科、外科等多学科的视角，融合了肺腺癌的部分新的免疫表型信息和分子信息，具有较高的临床实用性。但随着临床实践的深化，WHO肺癌分类体系越来越显现出其自身的局限性，主要表现在：肿瘤形态组织分类复杂，可重复性差；依据免疫标记将肺癌分为鳞癌和腺癌两大类，简单化的同时不可避免地出现片面性，部分肿瘤细胞同时表达鳞癌和腺癌的标记物，难做出明确的区分；WHO分类提供了有限的组织类型-分子表型信息；以上几点导致WHO新分类指导临床实践的效能大打折扣。

我国著名病理学家李维华教授曾提出以组织发生（来自支气管表面上皮、细支气管肺泡上皮、神经内分泌、支气管腺体）和组织表型（鳞癌、腺癌、神经内分泌癌）为基础，联系分化程度和结合免疫组织化学及超微结构特征的肺癌分化表型分类方案^[7]^。该分类方案体现了简明、系统和高度重复率的优点。Yatabe^[8]^于2002年提出了终末呼吸单位（terminal respiratory unit, TRU）的概念，并在此概念指导下，根据组织起源不同将肺癌分为TRU型和非TRU型，两型腺癌在临床特点、组织结构、免疫组化和基因突变等方面均有不同的特点，但该研究并未突出组织表型这一要素。

组织发生部位是肿瘤的重要特征之一，主支气管和肺由原基（肺芽）向各级气管及肺泡分化的过程中存在特定表型特征的逐步关闭和表达，而这种变化又和部位存在一定关联性。如在胚胎早期TTF-1的表达可见于大气管的假复层纤毛柱状上皮中，而在发育成熟的个体中仅见于肺泡单位和远端呼吸性细支气管中，可见肺癌的组织发生部位或组织起源代表了肺癌的表型和分化特征^[9]^。本研究中不同组织起源的肺癌与相对应的正常肺组织免疫表型呈现高度的一致性；同时单因素生存分析显示，临床Ⅰ期和Ⅱ期的细支气管肺泡癌患者的预后趋势优于支气管上皮癌但低于肺泡细胞癌，因为大多数的患者在术后接受了放化疗或靶向治疗，目前基于顺铂为主的联合治疗方法多用于NSCLC患者不同阶段的治疗^[10]^。不同的临床预后也从另一方面反映出不同起源NSCLC对于治疗方案的选择存在明显差异。以上分析表明，本研究提出的肺癌分类不但具有很高的病理分类诊断价值，也具有重要的临床治疗指导意义。

表皮生长因子受体酪氨酸激酶抑制剂已被广泛应用于*EGFR*基因突变型的NSCLC^[11, 12]^，并且已作为一线标准治疗药物列入相关治疗指南^[13, 14]^。本研究还发现，*EGFR*基因在支气管上皮癌、细支气管肺泡癌及肺泡细胞癌中的突变率分别为1.60%、52.30%及45.50%，差异有统计学意义（*P* < 0.001），反映了本分类体系下肿瘤类型与分子表型具有较高的一致性；同时不同组织起源的NSCLC中*EGFR*突变位点呈现不同特点，以上具有重要的临床提示意义，可以减少肺癌患者不必要的特定驱动基因的检测，降低患者的医疗费用和负担；同时有利于重点筛查出适合靶向治疗的患者，提高靶向治疗的精准度。

总之，本研究中提出的基于组织起源的NSCLC的分类可以提高临床病理诊治疗和有效指导临床治疗，具有充分的理论意义和临床实践价值。
